# A Direct Observation of Infanticide by a Female Free‐Ranging Dog (*Canis familiaris*) Supports the Resource Competition Hypothesis

**DOI:** 10.1002/ece3.72727

**Published:** 2025-12-28

**Authors:** Melissa Vanderheyden, Brenda Chaignon, Clément Car, Małgorzata Pilot, Ikhlass el Berbri, Sarah Marshall‐Pescini, Friederike Range, Andreas Berghänel

**Affiliations:** ^1^ Zoology and Animal Ecology Research Group, Department of Biology University of Hildesheim Hildesheim Germany; ^2^ Domestication Lab, Konrad Lorenz Institute of Ethology University of Veterinary Medicine Vienna Vienna Austria; ^3^ Department of Biology, Chair of Developmental Biology Friedrich‐Alexander‐University Erlangen‐Nürnberg Erlangen Germany; ^4^ Faculty of Biology, University of Gdańsk Gdańsk Poland; ^5^ Department of Pathology and Veterinary Public Health Agronomic and Veterinary Institute Hassan II Rabat Morocco

**Keywords:** Canidae, communal breeding, reproductive conflict, reproductive suppression, reproductive synchrony, threat of infanticide

## Abstract

Infanticide by females occurs in many mammalian species but is rarely observed directly, making its social and ecological determinants difficult to identify. Here, we report the first direct observation of within‐pack infanticide by a female free‐ranging domestic dog. To assess whether this case can be better explained by resource competition or nutritional gain, we examined the social context in which it occurred. In our long‐term study population, we identified 26 cases characterised by a similar pattern of overlapping pregnancies within social groups. By comparing dominance rank and birth order across cases, we found that subordinate females time their births after or in synchrony with the alpha female, which may be a strategy to reduce infanticide risk. By giving birth later, the subordinate potentially allows the alpha a competitive advantage, thereby reducing the incentive for infanticide. Furthermore, subordinate free‐ranging dogs may use communal denning as a protective measure, as it hinders the identification of individual litters. In our study, the infanticide followed a period of rank instability. Social rank instability could elevate infanticide risk, since the absence of dominance signals might complicate the subordinate's assessment of the potential threat to its offspring, or because infanticide is used to consolidate dominance. On the other hand, we did not find support for the hypothesis that insufficient nutrition motivated the infanticide, based on an evaluation of the body condition scores within the pack and on the observation that not all killed pups were eaten. Taken together, our preliminary findings suggest that resource competition rather than nutritional gain may better explain infanticide by female free‐ranging dogs.

## Graphical abstract

Infanticide by female mammals is rarely observed directly, making its social and ecological determinants difficult to identify. We report the first observation of a within‐pack infanticide by a female free‐ranging domestic dog and compare its social context with similar constellations across our study population. Our findings suggest that the infanticide was likely driven by resource competition rather than nutritional gain and that subordinate females may use protective strategies.

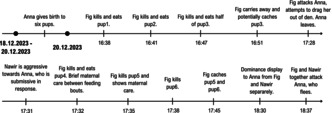

## 1 Introduction

Infanticide, here defined as ‘an act by one or more non‐parents that makes a direct or significant contribution to the immediate or imminent death of a conspecific's young’ (Digby 2000), has been studied more extensively in male mammals than in females. Decades of field research show that for males, infanticide has evolved in response to mating competition, as a strategy to create reproductive opportunities (Blaffer Hrdy 1974; van Schaik and Janson 2000; Hrdy 1979). While in some species, infanticide by females was observed more frequently than infanticide by males (e.g., in rabbits, hyenas, and African striped mice [Brown et al. 2021; Rödel et al. 2008; Schradin et al. 2009]), direct observations are generally rare and the determinants of infanticide by females are less well known, reflecting the historical bias towards studying male rather than female reproductive competition (Clutton‐Brock and Huchard 2013; Stockley and Bro‐Jørgensen 2011).

In a comparative analysis, Lukas and Huchard (2019) reported that infanticide by females has been observed in 89 out of the 289 mammalian species included in their sample. Their results show that female infanticide is more common in group‐living species, that high levels of relatedness do not necessarily have a protective effect, and that female infanticide is more likely motivated by preventing resource competition with the killer's offspring than by exploitation (i.e., consumption solely for nutritional gain).

The resources a killer safeguards for her offspring can vary. For instance, she may free up breeding space by killing infants outside her home range (e.g., black‐tailed prairie dogs [Hoogland 1985]), preserve access to her milk by killing unrelated pups that attempt to suckle (e.g., pinnipeds [Digby 2000]), or secure her offspring's future social status in nepotistic species where additional offspring help to strengthen the matriline (e.g., hyenas [Brown et al. 2021]). That infanticides are often a strategy to reduce competition between the respective offspring is additionally suggested by the findings that female killers are usually pregnant or have dependent offspring, and that under natural conditions, infanticide occurs more frequently in species that live in harsh environments (Lukas and Huchard 2019).

### 1.1 Infanticide in Canids

In the majority of grey wolf packs ( *Canis lupus* ), only a single female produces a litter (Mech 2025). Reproductive suppression appears to be behavioural rather than hormonal, as levels of luteinising hormone (LH), progesterone, and oestrogen are comparable in subordinate and dominant captive females, and ovulation occurs in both (Packard et al. 1985; Seal et al. 1979). Additionally, Sands and Creel (2004) show that reproductive suppression among wolves in Yellowstone National Park is not physiologically regulated through chronically elevated levels of adrenal glucocorticoid induced by social stress. Behavioural suppression varies in captive wolves; whilst some dominant females assault and intimidate subordinates during the mating period (Altmann 1987; Rabb et al. 1967), others merely increase their dominance displays during this time (Derix et al. 1993).

Under specific circumstances, suppression is less strict and multiple female wolves reproduce. Generally, breeding by multiple females is thought to be associated with surplus food (Mech and Boitani 2003). Studies have shown that wolf packs are more likely to have multiple breeders in areas with larger prey (Mech 2025) and under high population density (Ausband 2018; Ausband and Mitchell 2021). The latter might be due to limited breeding opportunities outside the group in saturated habitats, which both lowers the motivation for dispersal and increases the inclusive fitness advantages of permitting daughters to breed within their natal group. The resulting larger group sizes make it harder for dominant individuals to fully suppress reproduction (Ausband 2018; Ausband and Mitchell 2021). In addition, when between‐group competition increases with population density, larger groups and thus plural breeding can be advantageous (Preuschoft and Schaik 2000). Mech (2025) used winter pack sizes of more than 10 members as an indicator of multiple breeding and estimated that less than 10%–15% of the packs contained more than one breeding female. Packs with multiple breeding females may, however, be more frequent than can be estimated based on winter pack size, if the cubs of subordinate females are killed (Packard and Mech 1980).

For dominants aiming to monopolise reproduction, infanticide is the last remaining option if reproduction could not be prevented at earlier stages. Direct observations in captive groups of dominant females killing a subordinate's pups exist for grey wolves (McLeod 1990), including Arctic wolves (Martina Lazzaroni, pers. com.). Klinghammer and Goodman (1987) report multiple infanticides by captive female grey wolves, but without mentioning dominance ranks and including cases where infanticide happened after caretakers presented a mother with another female's pups, which they hoped she would foster. In other group‐living canid species, infanticide by the dominant female has been observed directly in captive dingoes ( *Canis dingo* ) (Corbett 1988) and in wild‐living African wild dogs ( *Lycaon pictus* ) (Creel 2022; Creel et al. 1997; Fuller et al. 1992; Girman et al. 1997). Circumstantial evidence, for example, finding dead pups without witnessing the killing, was reported in captive wolves (Altmann 1987; Rabb et al. 1967) and in wild‐living Ethiopian wolves ( *Canis simensis* ) (Sillero‐Zubiri et al. 1996). In African wild dogs, Frame et al. (1979) observed dominant females interfering with the provisioning of pups born to subordinates, causing the death of five of the six subordinate litters they studied. In many of these reports, the dominant female was either pregnant or nursing her own offspring when the subordinate's litter died (Altmann 1987; Rabb et al. 1967; McLeod 1990; Klinghammer and Goodman 1987; Corbett 1988; Creel 2022; Girman et al. 1997; Frame et al. 1979).

### 1.2 Infanticide in Free‐Ranging Dogs

Free‐ranging dogs show significant variation in their social structure, with some individuals living solitary, others in pairs or packs with up to 27 individuals (Bonanni and Cafazzo 2014). Mating is predominantly polygynandrous, with both males and females preferring high‐ranking partners (Cafazzo et al. 2014; Ghosh et al. 1984; Natoli et al. 2021; Car et al. 2025). Females can reproduce twice a year (Boitani et al. 2006), share the same home range with other breeding females, and benefit from alloparental care (Daniels and Bekoff 1989; Pal 2005). No strong reproductive seasonality was found (Cafazzo et al. 2014; Daniels and Bekoff 1989), except in Indian populations where most births took place in winter, outside the monsoon season (Oppenheimer and Oppenheimer 1975; Pal 2003).

Reports of adult free‐ranging dogs killing pups are rare (Pal 2005, 2001). Pal (2001) observed a mother killing and eating two of her already ill and dying pups, and adult dogs (sex not mentioned) killing and eating eight pups from two litters that did not belong to their own group. These killings accounted for 4.8% of the causes of death reported for 205 pups (Pal 2001). ‘Predation by adult dogs’ was mentioned as the cause of mortality for two of 35 pups studied by Pal (2005), without detailing the precise circumstances or the sex of the adults. Additionally, the protection of pups against strangers by both mothers and potential fathers (Pal 2005), as well as pack splitting (Daniels and Bekoff 1989), have been interpreted as potential preventive measures against infanticide.

Since infanticides happen rapidly and infrequently, direct observations of female infanticide are rare (Lukas and Huchard 2019). We contribute to the existing body of case reports by describing a direct observation of a within‐group infanticide by a female free‐ranging dog. Since the victims' mother gave birth at a time when both other female group members were pregnant, we hypothesised that the killing might be related to this particular social setting. Hence, we searched within our population for reference cases where a female gave birth during the pregnancy of one or more other pack members and identified 26 cases, including the infanticide case. Notably, in three of these cases the entire litter disappeared without a clear cause. By comparing birth order, dominance, and relatedness across these cases, we aimed to better understand how the social settings in which the infanticide and the disappearances occurred differed from the reference cases, to consider the potential drivers of the event, and to guide future research. In addition to this comparison, we investigated the stability of the dominance hierarchy and the nutritional status of the females involved in the infanticide case. Using these descriptive data, we explore two alternative hypotheses: the ‘exploitation hypothesis’ and the ‘resource competition hypothesis’. The ‘exploitation hypothesis’ predicts that infanticide occurs for nutritional gain; hence, the killer eats its victims and the killing occurs independently of the victim's age or the killer's reproductive state (Lukas and Huchard 2019). The ‘resource competition hypothesis’ predicts that females commit infanticide to reduce current or future competition for their own progeny. Hence, infanticides should be carried out by dominant females that are either gestating, lactating, or caring for dependent offspring and should target victims of any age or, in case of competition for allomaternal care, dependent offspring of closely related females (i.e., the typical competitors for and providers of allomaternal care) (Lukas and Huchard 2019; Vanderheyden et al. In preparation).

## 2 Methods

### 2.1 Observational Data Collection

A total of 1129 h of focal data were collected for 38 adult females between 15 March 2023, and 19 March 2024. The infanticide was observed on 20 December 2023, and fully recorded on camera (Panasonic HD‐Camcorder HC‐V180) (Video 1). The focal individuals, including the females involved in the infanticide case, belonged to eight stable social groups (Data S5) in our study area in Tamraght, a coastal town in the Souss‐Massa region, Morocco (30°31′N, 9°41′W). The individuals belonged to a stable continuous population that we have monitored since 2016 (see Berghänel et al. 2025). Monthly temperatures in the region vary between average maxima of 20°C–29°C and average minima of 11°C–20°C. The average monthly precipitation from October to March is 19 mm, with near‐zero rainfall during summer months. The dogs in the study area scavenge on human‐generated food sources and most are habituated to people, as they receive food from and interact with both tourists and locals. Genetically, our population clusters closer to other free‐ranging dog populations than to purebred dogs (Range and Marshall‐Pescini 2022), indicating that the population derived from a continuous lineage of free‐breeding dogs rather than an admixture of breeds, similar to Eurasian free‐ranging dog populations (Pilot et al. 2015). Rabies rarely occurred during the period included in this study, with to our knowledge only a single suspected case in the general population and no cases among the focal individuals.

**VIDEO 1:** Full video of the infanticide, recorded on 20 December 2024 by Brenda Chaignon. Video content can be viewed at https://onlinelibrary.wiley.com/doi/10.1002/ece3.72727.

We mainly focused observation efforts on the hours after sunrise and before sunset, when the dogs were most active. Each focal follow lasted 15 min, during which all social interactions (e.g., affiliative and agonistic behaviours) involving the focal individual were recorded. In addition to focal data, we recorded ad libitum all dominance, aggressive and submissive behaviours between all individuals. At the start of each focal session, physical condition was assessed using scores between 1 and 9, developed for veterinary assessments of pet dogs. Increasing scores represent increasing body fat deposits and thus body fat reserves and obesity, and the ideal score is 5 (Mawby et al. 2004). We also recorded whether a female was in heat, pregnant, or lactating, and the number of dependent pups. Pregnancy was also recorded via increasing body condition scores.

### 2.2 Reference Case Selection

We used the criterion of ‘births that took place when one or more pack members were gravid’ to select cases of birth succession from the litters that were born to focal females. The overlap was assessed based on a reported gestation length of 62 days in pet dogs (Concannon 2011), which matches with the median duration between parturition and the last observed day of heat in our population (59 days, IQR: 55.5–63.0, *N* = 83). In total we selected 26 cases in which litters were born during a group member's gestation period. This number includes the infanticide case and three cases in which an entire litter disappeared in a single event, within days of birth, when no observer was present. In the other cases, pup mortality was not linked to infanticide since one or more of the following characteristics were present: pup health declined during the days leading up to its death; the corpse was found without physical signs indicative of aggression; or pups died/disappeared one at a time rather than simultaneously. In a single case (referred to as ‘cheese_2’), a local resident culled three litters 36, 40 and 45 days after birth and communicated this to us afterwards.

### 2.3 Dominance Hierarchy

Dominance relationships were assessed in R (R Core Team 2024) using a Bayesian adaptation of the Elo‐rating method (Elo 1978) with the R package EloRating.Bayes (Neumann 2025). One‐sided submissions, either spontaneous or following an agonistic interaction, and one‐sided greetings were treated as decided wins. In addition, the program takes undecided interactions into account but assigns them less weight. Greetings, defined as affiliative interactions whereby one individual licks or pushes with its nose the recipient's muzzle, were included as a formal submissive signal since they show high directional consistency and were found to covary with other selected measures of agonistic rank in both domestic dogs (Cafazzo et al. 2010; van der Borg et al. 2015) and Arctic wolves (Cafazzo et al. 2016). To reflect the differences in interaction types (Newton‐Fisher 2017), we divided behaviours into greeting, weak submission (e.g., avoid, avert gaze), and strong submission (e.g., crouch, belly exposure), and estimated separate k‐values for each type to assign them different impacts on the hierarchy.

Classic Elo‐rating methods either use the same start score for all individuals, allowing for differentiation only after a burn‐in period, or need previously collected data to determine individualised start values. EloRating.Bayes, however, allows for the estimation of the start values by using the available data as a prior. Therefore, dyads with a stable relationship often already start with the rank relationship they maintain throughout the observation period.

We calculated the dominance relationships between the females in the pack where the infanticide took place on the day of the event. For 8 out of the 15 birth sequences used as reference cases, the dominance hierarchy among female group members was calculated on the day the first female gave birth. Instances where this was not possible included two where the first birth took place shortly (16 and 19 days) after the end of the focal observations. For those, we calculated the hierarchy on the last day of data collection.

In five cases, the first birth took place before (*N* = 2) or within (*N* = 3) the first months of the data collection period, hence no or limited data were available for rank calculations on the first birth date. To ensure reliable rank order estimation, we calculated dominance hierarchies for dates when each individual had accumulated at least five dominance interactions within the pack. When individuals died before they accumulated five interactions, which happened in two cases, the dates when the others in the packs reached this threshold were used, respectively 0 and 4 days after the first birth dates. For one of the cases where a female died before having five interactions, we included the entire social group in the analysis as there had been no interactions between the females themselves. We believe this result to be accurate as it shows that the mother, who was at least 4 years old, ranked six positions higher than her one‐year‐old daughter, which aligns with previously reported patterns of dominance in free‐ranging dogs (Cafazzo et al. 2010; Bonanni et al. 2017). Additionally, the start values assessed by the EloRating.Bayes function based on all interactions during the entire period (see above) implied the same rank order.

In the cases where all females survived until they reached the threshold, the hierarchies were calculated 33, 70, and 138 days after the first birth. While the 138‐day gap was large, we trusted the accuracy of the involved females' ranks since they never changed their dyadic dominance relationship during the data collection period of 12 months. The relationship between the females who had the 70‐day gap was less clear‐cut at the start of the observation period as the 90% credible intervals overlapped significantly for some time. However, the hierarchy became more pronounced later, confirming the order we found for the calculation date, where the mother outranked her daughter.

### 2.4 DNA Collection and Analysis

We collected saliva samples from all 26 individuals included in this report, using noninvasive collection kits (Performagene PG 100, DNA Genotek, Canada). The DNA was extracted according to the manufacturer's instructions. SNP genotyping was conducted using the Axiom Canine HD Genotyping Array (Thermo Scientific). For 15 individuals (Table S1), DNA extraction and genotyping were successful, generating a dataset of 201,723 autosomal SNPs with less than 10% missing data. Plink1.9 software was used for data filtering (Chang et al. 2015) and to estimate relatedness using the pairwise identity by descent (IBD) coefficient pi‐hat, referred to in the text as ‘kinship coefficient’. For a full description of the methods of genetic kinship reconstruction, see Berghänel et al. (2025).

### 2.5 Statistics

To examine whether subordinate females were less likely to give birth before a dominant female, we fitted Bayesian intercept‐only binomial models with logit link function, using R packages brms and tidybayes (Bürkner 2017; Kay 2024). Each data point consisted of a dyad of mothers who belonged to the same group and gave birth within 62 days of each other to ensure overlapping pregnancies, following gestation lengths reported for pet dogs (Concannon 2011). The response variable was dyadic and coded 0 if the subordinate gave birth before the dominant female, and 1 if the subordinate's parturition occurred on the same day or after the dominant. To account for repeated measures, we included individual 1 and 2 as a combined multi‐membership random intercept and case (Figure 3) as a random intercept. We implemented multi‐membership models to address the dyadic structure of the data, meaning that individuals can appear as either individual 1 or individual 2 of a dyad.

This model structure allowed us to test whether, at the population level, the probability that a subordinate female gives birth on the same day or after a dominant female was above chance (intercept > probability of 0.5, corresponding to a logit value of 0). We ran three versions of the model: one including all dyads, one only including dyads where the dominant was an alpha female and one only including dyads where the dominant was not an alpha female. All models used 4 chains with 2000 iterations, 1000 warmup, and 4000 post‐warmup draws. Delta was set to 0.95 throughout to avoid divergent transitions.

Since this is a single case study, additional statistics were mostly descriptive.

## 3 Results

### 3.1 Involved Individuals

Originally, the pack where the infanticide occurred included four adult females: Groom, Fig, Anna, and Nawir. The alpha female, Groom, disappeared one and a half months before the infanticide. In 2019, Groom cared for a group of five sub‐adults, estimated to be younger than six months, including Fig and Nawir (Giulia Cimarelli, pers. com.). Kinship data were successfully obtained for Nawir and two of the other juveniles, confirming them to be full siblings. It is therefore likely that Fig and Nawir are at least half‐siblings (multiple paternity has been observed in free‐ranging domestic dogs [Natoli et al. 2021], including our study population [Car et al. 2025]), although genetic data for Fig or Groom were not available. Genetic evidence identified Anna as Nawir's daughter, implying that she is likely Fig's niece. At the time of the infanticide, Fig was the new alpha female, with Nawir ranking second and Anna occupying the lowest rank (see Section 3.5). Both Fig and Nawir were pregnant.

Several adult males were present regularly, albeit less consistently than the females. Three of them, Medo, Date, and Hazel, were at the time present within 50 m of the infanticide scene. Hazel is Anna's full sibling. The other males' relatedness to the females and the killed pups is unknown since we do not have genetic data for Anna's pups, Medo, or Date. The latter two were seen around Anna when she was in heat, together with six other males from the same or neighbouring packs.

**FIGURE 1:**
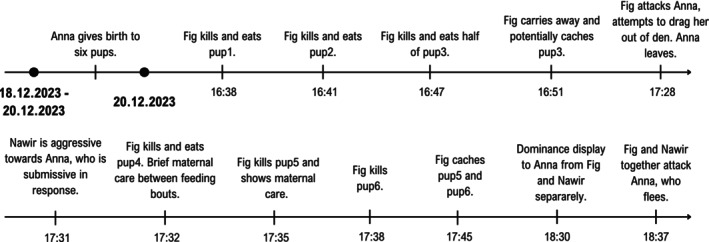
The timeline of the infanticide.

On 20 December 2023, Fig killed all six of Anna's newborn pups. The incident took about an hour and ten minutes (Figure 1, Video 1). None of the males interfered with the killing.

### 3.2 Timeline of the Infanticide

On 18 December 2023, Anna was observed at 08:00, still heavily pregnant.

**FIGURE 2:**
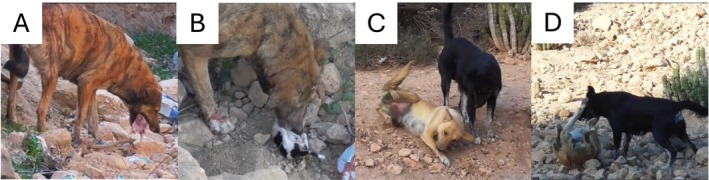
(A) Fig consuming pup3 and (B) licking pup5 on the belly and anogenital area. (C, D) Aggression from Nawir (right) and submission from Anna (left).

On 20 December at 16:28, Anna was seen lactating. Ten minutes later, Fig put her head inside the den, which contained Anna and her newborn pups. Anna growled from inside the den but did not intervene when Fig took away pup1. Fig killed and ate the pup four meters in front of the den, starting with a decapitation (Figure 2a). At 16:41, Fig returned to the den and removed pup2 whilst both females growled at each other. In the same location and using decapitation as before, Fig killed and ate the pup. When Fig returned to the den at 16:47, she responded to Anna's growling by attacking her. Details of the attack could not be observed as it happened inside the den at an angle that prevented the observer from looking inside, but it sounded like Fig bit Anna, who whimpered loudly. Next, she removed pup3 and killed it as before. After eating only half of it, she rolled on the corpse, carried it away and cached it about 15 m from the den. She returned to the den at 17:08, was met with growling from Anna, retreated and remained at five meters from the den where Anna continued to nurse her pups.

At 17.28, Fig approached the den again and entered with the head. After mutual growling and barking, Fig attacked Anna. She bit into Anna's cheek, held her there and tried to pull her out of the den, which partly collapsed. Anna then submitted and retreated, leaving the den with her three remaining pups unprotected. When at 17:30 Medo, a male group member, approached within five meters without showing aggression, Fig growled at him. At 17:31 and about 20 m from the den, Anna submissively approached Nawir, who started growling. In response, Anna crouched and rolled onto her back in submission. As Anna tried to get back up, Nawir attacked her, biting her briefly and superficially at the neck (Figure 2c,d). Fig carried pup4 out of the den at 17:32, to the same spot where she killed its siblings. She bit off and ate the head, then briefly performed anogenital licking, normally a maternal behaviour, and ate the rest of the body. Meanwhile, Nawir kept growling at Anna, who crouched and exposed her belly in response. Medo marked at about five meters from Nawir and Anna, and about nine meters from Fig, witnessed the aggression from Nawir to Anna, but did not interact with any of the females. At 17:35, Fig carried pup5 out of the den, again to the same location. The pup did not move and was likely already dead. Like pup4, she licked the pup in the anogenital area and on the belly (Figure 2b). Without consuming pup5, Fig left the corpse behind to return to the den at 17:38. She removed the last pup, pup6, from the den, carried it to the usual location, decapitated it and ate only the head. At 17:45, Fig cached the last two corpses in different locations, at about 40 and 50 m from the den, respectively.

At 18:28, Anna, Fig, Medo, Hazel, and Date barked and lunged together at either a human or an unknown dog, but it was not possible to see their target. At 18:30, Anna approached the location where Fig had cached the corpse of pup6. Fig approached Anna at the caching site. She stood tall and growled at Anna, who submitted by lowering her tail and rolling onto her back. Anna left Fig and was approached by Nawir, who stood tall and growled at her. She responded by crouching and exposing her belly. At 18:37, Fig approached Nawir, and they attacked Anna together. Anna first submissively exposed her belly, then fled and hid in a shrub (within 50 m of the den).

On 21 December, the day following the infanticide, Anna was the only female of the pack that could be found in the area. She did not show any unusual behaviour during the observation. Medo and Hazel were seen within 10 m of her. All females were found in their usual location on 22 December and no unusual behaviour was observed. In the days and weeks following the infanticide, there was no noticeable difference in the behaviour of the involved individuals.

### 3.3 Birth Order and Litter Disappearance

**FIGURE 3:**
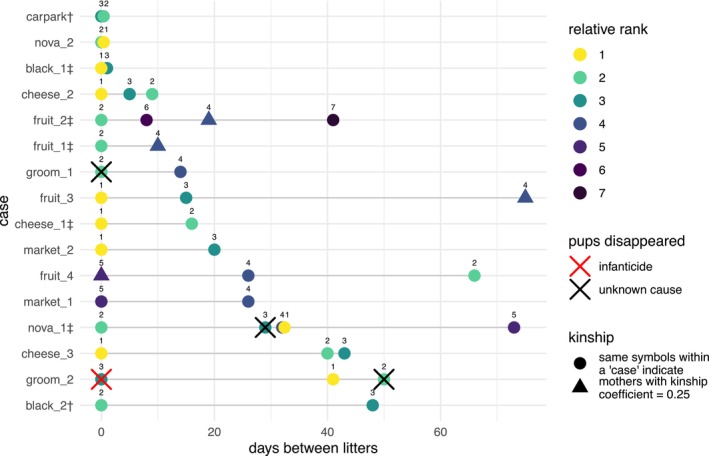
The parturition dates and relative dominance ranks for cases where female group mates had overlapping birth and pregnancy dates, with the first litter of each case born on day 0. Colours indicate relative dominance ranks within the females of the group. Identical symbols show whether the individuals within each case are related with a kinship coefficient of 0.25 or higher (e.g., half‐siblings). Kinship values are based on genetic data where available (*N* = 15), otherwise on detailed long‐term demographic data (*N* = 10). Symbols next to the case names indicate whether dominance ranks were calculated before (†) and after (‡) the first birth date (see Section 2). Case names consist of the group name and a case number. Multiple cases within the same group do not always include the same females.

**FIGURE 4:**
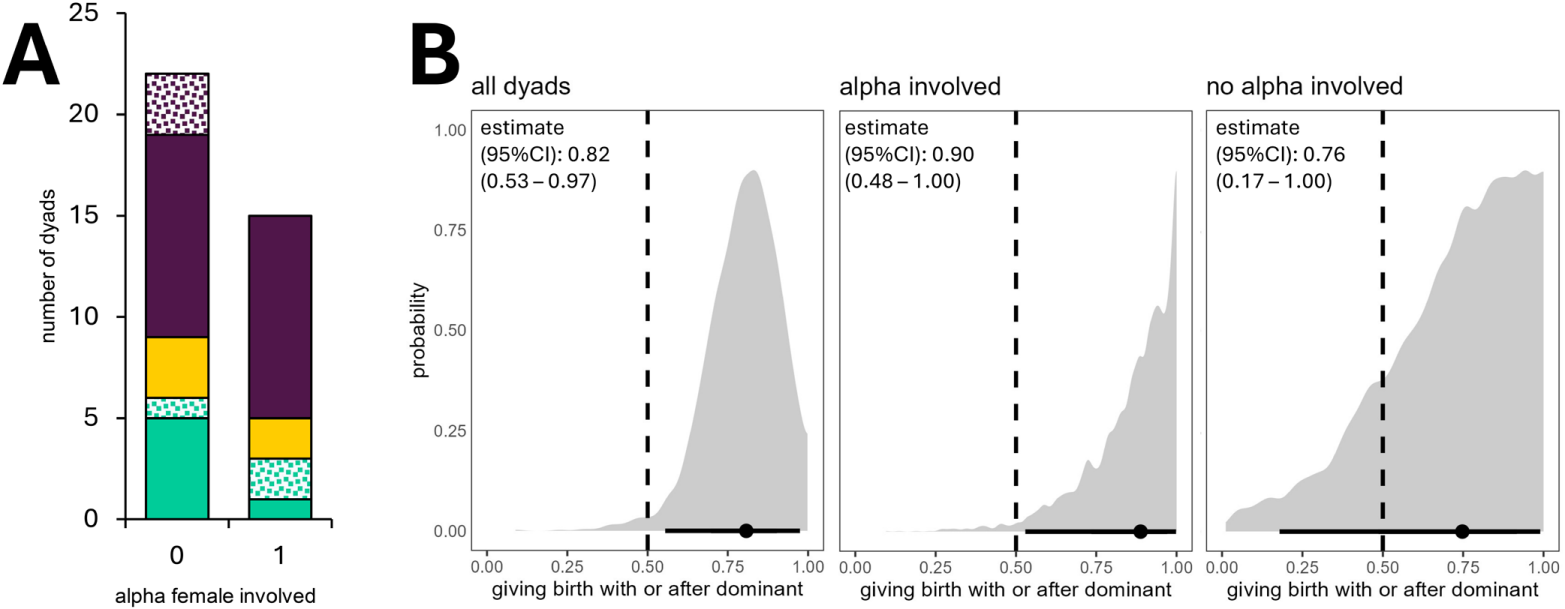
Subordinate females tended to give birth later than or on the same day as dominant females, and this effect was more pronounced if the dominant was the alpha female. (A) In dyadic birth successions, subordinate females more frequently gave birth *after* (purple) or *on the same day as* (yellow) more dominant females, rather than *before* them (green). This difference was more pronounced in dyads where the more dominant female was the alpha. Dotted areas represent all dyads which included a litter that disappeared or was killed. (B) Posterior distributions from Bayesian binomial intercept‐only models, showing the probability that subordinate females gave birth on the same day as or after a dominant female (back‐transformed results, dotted line at 0.5 reflects 50% chance level). Bottom point and line: Median intercept and 95% credible interval (CI).

On the date of the infanticide, both other female group members were in the early stages of pregnancy. Fig gave birth 41 and Nawir 50 days later, with their heat periods ending 14 and 10 days before the infanticide, respectively. Among all selected cases of overlapping pregnancies in focal females, births often occurred in clusters, occasionally leading to sequences of more than two overlapping pregnancies within the same group (Figure 3, Data S6). Altogether, the dataset included 16 such clusters or successions, involving 26 females across seven packs, which resulted in 37 dyadic combinations of overlapping pregnancies within the pack (Figures 3 and 4A). We compared these birth successions, aiming to understand the context in which the infanticide and other litter disappearances took place.

In a dyadic analysis, subordinate females generally gave birth later than or on the same day as more dominant females, and this effect was especially pronounced when the dominant was the alpha female (Figure 4, Data S7). The binomial intercept‐only models including all dyads (*N* = 37) showed a 98.0% probability of subordinate females giving birth on the same day as or after the dominant female, with a posterior median of the intercept [95% CI] of 0.82 [0.53–0.97] (back‐transformed to probability scale). The intercept represents the estimated proportion of subordinate females giving birth on the same day as or after the dominant female, after accounting for random effects and repeated measures. For dyads in which the dominant female was also the alpha female (*N* = 15), the probability of subordinates giving birth on the same day or after the alpha was 97.4%, with a higher posterior median of the intercept [95% CI] of 0.90 [0.48–1.00]. In contrast, for dyads where the dominant was not the alpha female (*N* = 22), the probability of subordinates giving birth on the same day or later was lower at 82.4%, with a median intercept [95% CI] of 0.76 [0.17–1.00]. However, the difference between the dyads with and without alpha was not significant (added binomial predictor variable: 0.66 [0.14–1.00]).

The alpha female was involved in 9 out of the 16 birth successions (Figure 3). In these 9 cases, 10 subordinates gave birth after the alpha and two on the exact same date. Out of three females who gave birth before the alpha female, two lost their entire litters early after birth. The first one is the mother whose pups were killed during the described infanticide (groom_2, Figure 3). In the second case, a female gave birth three days before two related group members (her mother and her (half‐)sister) delivered their litters, and 44 days before another (half‐)sister gave birth (nova_1, Figure 3). Her mother, the dominant female, was seen to inspect the den from the outside on the day the litter was born. Within a day, the pups had disappeared and the den collapsed. Direct observations of how they disappeared are absent. No traces of blood were found near the den, whilst the infanticide did leave some visible blood marks at the killing spot.

Litters disappeared in two additional cases, both involving the females of the pack where the infanticide took place. In the first case (groom_1, Figure 3), Fig gave birth on 12 August 2023, to pups that were not seen after the initial observation on the day of their birth. Her mother Groom was still alive and the alpha female at the time but did not reproduce that year. The second case (groom_2, Figure 3) concerns Nawir, the female ranking below Fig, who gave birth a month after the infanticide, on 8 February 2024. In both cases, the pups disappeared within days after they were born, and no traces of blood were found. During the single focal observation of Nawir with her pups, Fig was seen whimpering in front of the den entrance.

Although no blood was found after any of these disappearances, traces might be difficult to detect in cases where the killer carried the pups away before killing them. In none of these cases did the mother disappear or abandon her pups, and she was seen in the territory of the social group in the days after the disappearance. Other potential explanations for disappearing litters are population control carried out by residents or predation by, for example, wild boars. In the past, our field team observed occasional conflicts between wild boars and free‐ranging dogs close to where the disappearances took place (Figure S4), and residents have communicated about the occurrence of culls (mostly of adult dogs or older pups) to us on a few occasions. However, assessing the likelihood of either scenario is difficult as we do not have recent observations of interactions with wild boars, and information about culls remains incomplete and unreliable.

### 3.4 Kinship

Genetic kinship data were obtained for 16 out of the 26 females involved in the cases of successive births. For six additional females, the genetic data from pups could be used to derive relatedness to other female pack members (see Table S1, Note S2, Data S12). For the remaining four females, kinship was inferred based on demographic data, that is, observations of presumed mothers grooming and/or nursing pups or juveniles. In 12 out of the 16 cases of successive births, or six out of the seven packs, all females involved were related at the level of half‐siblings or higher (kinship coefficient ≥ 0.25). In all four instances of pups disappearing within days of their birth, including the observed infanticide, the mother was closely related to the other group members. In the two cases where an unrelated subordinate gave birth before a dominant female, the litters survived. This suggests that the risk of infanticide is not necessarily lower among kin.

### 3.5 Dominance Instability

Overall, the dominance hierarchies were reasonably stable during the observation period, with mothers outranking their daughters (Figures S3A–G and Data S9–S11). Alpha females outranked all female group mates for the majority of the time, except in one pack where the second‐ranking female's dominance scores were similar to the alpha female's for limited periods of time, although she generally ranked lower (Figure S3D). Where consistently unclear rank‐relationships occurred, they usually involved siblings.

**FIGURE 5:**
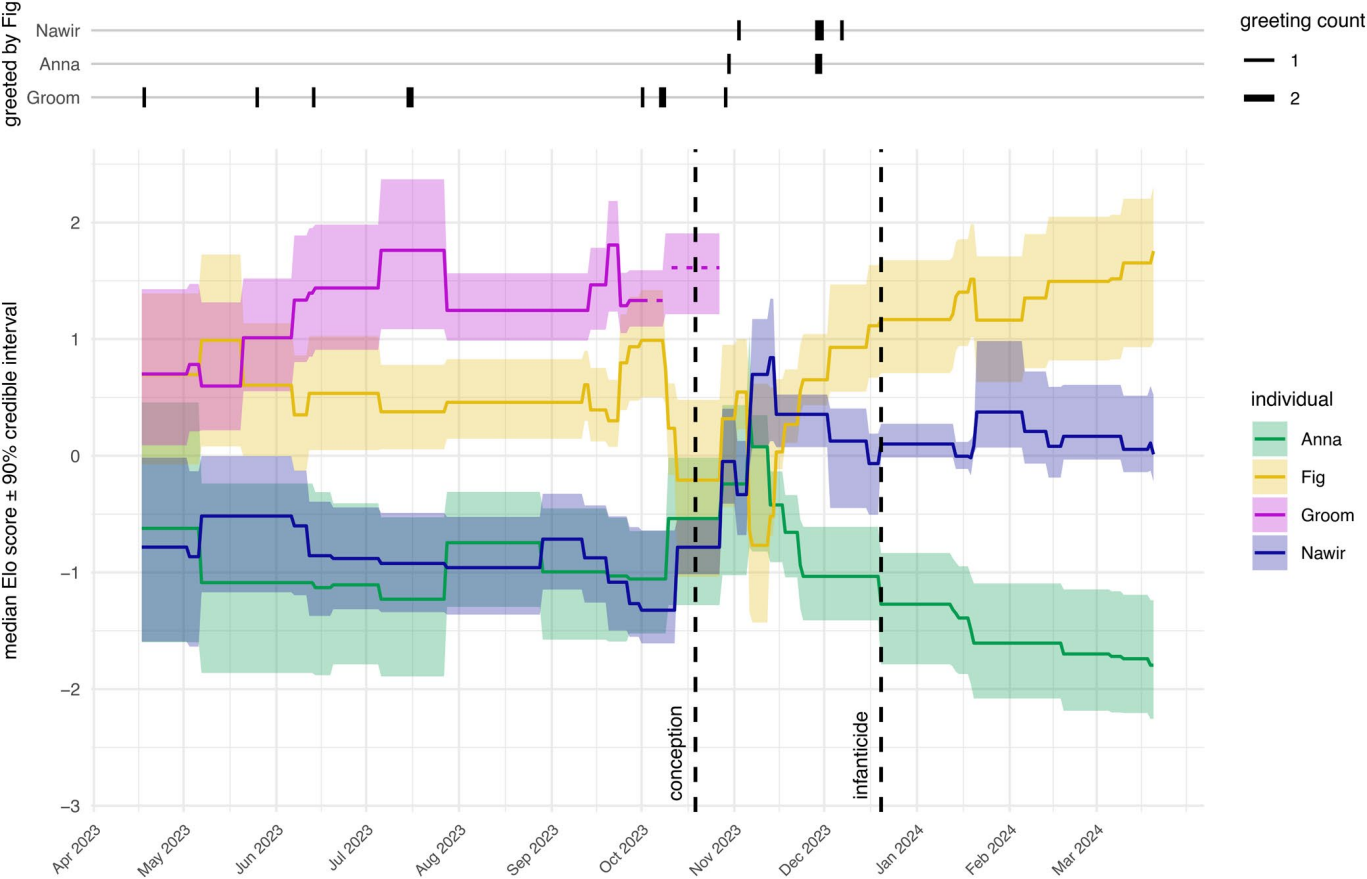
Bottom: Median Bayesian Elo‐ratings with their 90% credible intervals for all females in the group where the infanticide was observed. Dashed black lines indicate the dates of the infanticide (1 or 2 days after birth) and of Anna's likely conception. Dashed lines in Groom's Elo‐ratings indicate the period when she was frequently absent, ending in her disappearance after 23 October. Top: Number of times Fig greeted the other females in the pack.

The only occasion where a high‐ranking individual's Elo scores took a large dive is in the pack where the infanticide took place during the period preceding the infanticide (Figure 5). At the time of the incident, Fig was the most dominant female, with Nawir ranking below her and Anna at the bottom. Mean posterior Elo scores before the infanticide were for Fig 1.13 (90% CI: 0.67–1.63), for Nawir −0.10 (90% CI: −0.50 to 0.19) and for Anna −1.03 (90% CI: −0.41 to −0.61) (Table S1). Although at that point in time the scores were differentiated enough to not overlap in their 90% credible intervals, they indicated instability in the hierarchy during the three months leading up to the event.

In October, the then‐dominant female Groom, presumably Fig and Nawir's mother (see above), was less frequently present in the group, which was unusual. The observers found her in locations she had never been seen before, sometimes accompanied by Fig. Groom eventually disappeared after 23 October. Around this time, Fig's Elo score dropped as a result of an increased number of greetings directed at Nawir and Anna, although Fig had never greeted either of them before (Figure 5, top). After January 2024, the greetings between Fig and the other two females again became purely unidirectional and directed at her, reflecting re‐established stability in the hierarchy. We estimated Anna to have conceived around 19 October 2023, which was the only day within her heat period (18 October– 28 October) for which an observed mating attempt (with Cheetah, a male group member who was not present at the time of the infanticide) confirmed her receptiveness. The resulting pregnancy duration of 62 days aligned with the average pregnancy duration in dogs between 62 and 65 days (Concannon 2011). Thus, the conception would have occurred when the hierarchy was unstable.

In the packs where the two independent litter disappearances took place, the Elo scores do not indicate instability or changes in hierarchy during the preceding weeks.

### 3.6 Body Condition Scores

**FIGURE 6:**
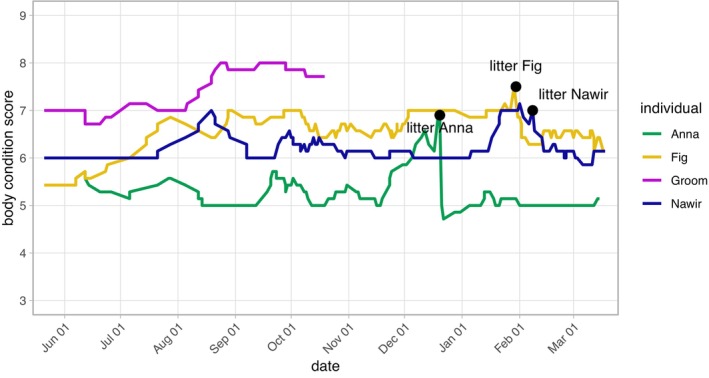
Individual body condition scores smoothed using a 7‐day‐centered rolling mean to minimise the influence of short‐term variability and observer bias. Pregnancy periods resulted in higher scores (see Section 2).

In the pack where the infanticide occurred, body condition scores were almost always above 5 (i.e., the ideal body condition) for all females, indicating that resource availability was sufficient (Figure 6, Data S8). Between the females, scores appeared to reflect dominance rank (Figure 5). The killer had the highest body condition score, and there was no obvious drop in scores in the period leading up to the infanticide.

## 4 Discussion

To our knowledge, this is the first directly observed case of within‐group infanticide by a female free‐ranging dog. The infanticide was carried out by the highest‐ranking female in the pack, who, based on observational demographic data, was likely related to the pups. It happened at a time when both the killer and the second‐ranking female were pregnant. Resemblances between these observations and documented cases of female infanticide in other canids indicate that this behaviour may be an ancestral trait that has been preserved in domestic dogs. Although some reports assumed a lack of dominance hierarchies in free‐ranging dog packs (Boitani et al. 2006; Boitani and Ciucci 1995; Van Kerkhove 2004; Bradshaw et al. 2009), partly based on the apparent absence of reproductive suppression of subordinate females, our results align with more recent studies that quantitatively analysed the frequency of one‐sided submissions and report stable dominance relationships within groups (Cafazzo et al. 2010; Bonanni et al. 2017; Bonanni and Cafazzo 2014).

### 4.1 Resource Competition

Our observations support resource competition as the primary cause of female infanticide in free‐ranging dogs by highlighting the influence of dominance rank and birth order on infanticide risk. This is in line with a large comparative study across mammals, which found that infanticide by females occurs when conspecific infants might limit access to resources that are critical for the killer's dependent offspring (Lukas and Huchard 2019). In wild canids, such critical resources likely include food, alloparental care (see below), social status and, depending on the habitat, denning sites (Davies et al. 2016). Our results on a population under high food abundance suggest that critical resources must be restricted or unpredictable over evolutionary timescales for female infanticide to evolve and occur, but not necessarily on a proximate level. Both in our observation as well as in reports of infanticide in grey wolves, dingoes and African wild dogs, the infanticide was carried out by the alpha female in the group, without significant interference from the victims' mother or other pack members (McLeod 1990; Corbett 1988; Creel et al. 1997; Fuller et al. 1992; Girman et al. 1997; Frame et al. 1979; Martina Lazzaroni pers. comm).

Our observations suggest that the threat of infanticide might have been higher for subordinates who gave birth when the alpha female was pregnant. In our population, subordinates who reproduced around the same time as the alpha female generally gave birth after her or on the same day. Moreover, out of the three subordinates who gave birth before the alpha female, one mother's litter was killed and another one's pups disappeared. Across species, birth order determines a litter's competitive abilities, with earlier‐born offspring typically being larger and thus stronger competitors (Dey et al. 2014; Hodge et al. 2009). By removing them, a female eliminates the competitive disadvantage of her later‐born offspring. Females have been observed to remove other females' offspring before, but not after, they breed themselves in communally breeding house mice ( *Mus domesticus* ) (König 1994), banded mongooses ( *Mungos mungo* ) (Gilchrist 2006; Hodge et al. 2011) and greater ani birds ( *Crotophaga major* ) (Riehl 2016). In the latter two, the selective pressures of resource competition and infanticide lead to strong birth synchrony.

After giving birth, infanticidal behaviour becomes risky if offspring are pooled and litters are difficult to distinguish (Altmann 1987). Hence, the behaviour might be inhibited through hormonal changes induced by birth and lactation (McCarthy 1990), preventing misdirected infanticide. In canids too, birth synchronisation and pooling of litters are used as strategies to counter infanticide. Studies on African wild dogs and captive wolves that include both infanticides and successful instances of communal breeding noted that subordinates who gave birth synchronously with the alpha female and pooled their litters with hers enhanced their offspring's survival (Altmann 1987; Creel and Creel 2002). In our population of free‐ranging dogs, these evolutionary pressures may also explain our observations of synchronised births and pooled litters within packs (Vanderheyden et al. In preparation).

### 4.2 Alloparental Care

In communal breeders, a potentially contested resource is alloparental care, and particularly allonursing. Allonursing has been observed in free‐ranging domestic dogs (Daniels and Bekoff 1989; Pal 2005; Pal et al. 2021; Paul and Bhadra 2017, 2018) and in all wild *Canis* species (Lord et al. 2013; Pecorella et al. 2023).

The hypothesis that competition for alloparental care drives infanticide in canids is supported by observations in grey wolves and dingoes, where the killer was usually the dominant breeding female who was pregnant or lactating in all but one case, and the victims were killed within days of birth, when still dependent on maternal care (Lukas and Huchard 2019). Moreover, the victim's mother sometimes allonursed the killer's pups after losing her own offspring (McLeod 1990; Corbett 1988). Birth order plays the same role in competition for alloparental care as it does for other food sources, and younger pups have lower chances of survival when competing for milk with older peers, for example, in brown rats ( *Rattus norvegicus* ) (Mennella et al. 1990). Hence, mainly earlier‐born litters can be expected to be targeted, especially since allonursing mainly happens in combination with communal denning, where the risk of accidentally killing own offspring would be higher.

The infanticide we observed did not entirely match this pattern, since the alpha female's litter was born too late (41 days) for the victims' mother to allonurse it. However, the second‐ranking female whose litter was born nine days after the alpha's was seen to guard and groom the alpha's pups after the disappearance of her own litter and while allonursing was not observed, dry suckling was.

### 4.3 Kinship

In our study, the killer was very likely the aunt of the victims' mother, which aligns with the finding that in infanticide by female mammals, kinship degree does not have a protective effect (Lukas and Huchard 2019). Among the other cases of overlapping parturitions in our study, females who were not related to dominant group members (four cases) were never reported to lose a litter, even if they gave birth before the dominant. Additionally, if the victim's mother is related to the killer, obtaining indirect benefits by nursing the killer's offspring may be her next best option.

### 4.4 Social Status

Infanticide may also result from competition for social status of the killer and/or her offspring. Social status as a driver of female infanticide has been associated with species that have nepotistic hierarchies and/or evict female group‐members (Lukas and Huchard 2019), aspects that have thus far not been thoroughly studied in free‐ranging dogs.

However, our observations indicate the social status may have played a role in the infanticide. Firstly, we observed strong dominance behaviour from the second‐ranking female towards the victims' mother. This has not been reported for infanticides in other canid species, but was observed in spotted hyenas ( *Crocuta crocuta* ), during infanticides which were likely driven by competition over status (Brown et al. 2021). In that study, Brown et al. (2021) describe how one or multiple group members attacked or chased the victim's mother, whilst her offspring was being killed.

Secondly, the infanticide we observed occurred in the context of the recent disappearance of the previous alpha female and the consolidation of the new alpha female in the dominance hierarchy. The interpretation of dominance consolidation as a driver for the infanticide may be further supported by the disappearance of the second‐ranking female's litter, despite her giving birth after the new alpha female. However, it is important to note that the circumstances surrounding this second disappearance remain unclear and may have been the result of external factors, as mentioned above.

Thirdly, the infanticide may have compensated for failed reproductive suppression. In other canids where female infanticide has been reported, the dominant female normally prevents subordinate female pack members from conceiving through behavioural (wolf: [Altmann 1987; Rabb et al. 1967; Derix et al. 1993]) or hormonal (African wild dogs: (Creel et al. 1997); Ethiopian wolves: [Van Kesteren et al. 2013]) means, indicating that infanticide might be a fallback option in these species. In our population, behavioural suppression was not as pronounced as described for wolves. However, subordinate females might forego reproduction or adjust the timing of parturition to the alpha female in the presence of clear dominance signals or cues if they indicate threat of infanticide, without the need for additional behavioural suppression by the dominant.

Importantly, for threat‐based reproductive suppression to be evolutionarily maintained, dominance signals (e.g., from the alpha female) must reliably predict an actual risk of infanticide; if suppression fails, the infanticide must be conducted. Otherwise, restraint from subordinates would have no adaptive value (Cant and Young 2013). This may also explain why proximate changes in resource abundance have only limited influence on infanticide propensity. For the infanticide we observed, it's relevant to consider the social constellation at the time the victims' mother conceived: the previous alpha female was frequently absent, triggering instability in the hierarchy. From the subordinate's viewpoint, self‐regulating reproduction only makes sense when the risk of infanticide and the associated cost are sufficiently high, which can't be assessed in the absence of threat signals or cues from the alpha female (Benvenuto and Lorenzi 2023). That absence of clear dominance cues or signals increases the chance of multiple breeding. This has been seen in wild canids when multiple breeding occurred after the loss of the dominant female in wolves (Ausband et al. 2017) and after shifts in the social hierarchy in red foxes ( *Vulpes vulpes* ) (Macdonald 1979).

### 4.5 Exploitation Hypothesis

In contrast to the competition hypothesis, the exploitation hypothesis argues that the primary driver of female infanticide is the nutritional gain from consuming the pups (Lukas and Huchard 2019). While nutritional gain might have offered added benefits, it is unlikely to be the primary motivation behind most reported infanticides in female canids. Free‐ranging dog pups have been reported to be predated by members of a different group (Pal 2005, 2001), and we observed a single case of pup predation by a (probably male) pet dog in our population. However, our reported case differs from these predations in that the killer was a related group member. She systematically killed all pups despite not fully consuming three of the victims, likely indicating satiety, but instead cached the corpses. Moreover, the strong dominance behaviour by both the second‐ranking female and the killer towards the victims' mother was never observed in a feeding context, even around valuable food sources. Additionally, in some reported cases of female infanticide in wolves, the victims were not eaten despite contextual similarities with our case, suggesting the same underlying primary motivation, unrelated to nutritional gains (McLeod 1990). In our population, it is not unusual for pups who died of natural causes to be eaten, often by the mother or siblings. Finally, all female pack members involved in the infanticide had body condition scores exceeding the ‘ideal’ for pet dogs (Mawby et al. 2004). The body condition scores followed the dominance hierarchy, suggesting contest competition, as perfect scramble competition would result in equal food intake for all individuals. Since the killer was the highest‐ranking female, her body condition score was the highest as well, suggesting slight to moderate obesity and making nutritional gain an unlikely motivator.

### 4.6 Limitations

Since our study reports only a single case of infanticide by a female free‐ranging dog, it is not possible to generalise and draw firm conclusions on the drivers of infanticide in this species. A specific challenge when studying free‐ranging dogs is that the disappearance of individuals can potentially be attributed to human influences, such as population control. Additionally, our most detailed observational data mainly stem from focal follows, which were only conducted for a single year.

## 5 Conclusion

To conclude, our detailed case study of an infanticide by a female free‐ranging dog involved patterns and behaviours that were similarly observed in wild canid species. The reported incidents of infanticide among the group‐living members of this species seem to be better explained by the resource competition hypothesis, with alloparental care as a likely influential resource than by the exploitation hypothesis.

## Author Contributions

**Melissa Vanderheyden:** conceptualisation (equal), data curation (equal), formal analysis (equal), investigation (equal), methodology (equal), writing – original draft (equal), writing – review and editing (equal). **Brenda Chaignon:** data curation (equal), investigation (equal), writing – review and editing (equal). **Clément Car:** formal analysis (equal), writing – review and editing (equal). **Małgorzata Pilot:** formal analysis (equal), funding acquisition (equal), writing – review and editing (equal). **Ikhlass el Berbri:** project administration (equal), writing – review and editing (equal). **Sarah Marshall‐Pescini:** funding acquisition (equal), project administration (equal), writing – review and editing (equal). **Friederike Range:** funding acquisition (equal), project administration (equal), resources (equal), writing – review and editing (equal). **Andreas Berghänel:** conceptualisation (equal), formal analysis (equal), funding acquisition (equal), methodology (equal), project administration (equal), supervision (equal), writing – review and editing (equal).

## Ethics Statement

Ethical approval for DNA sampling was obtained from the Ethical Committee for Animal Veterinary Science and Public Health of the University Hassan II Rabat, Morocco (Protocol number: CESASPV_2023_04). All procedures applied in the present study were noninvasive and in accordance with the European Union Directive on the protection of animals used for scientific purposes (EU Directive 2010/63/EU). For behavioural observations, the Research Ethics Committee of the University of Veterinarian Medicine Vienna has confirmed that no ethical approval is required. Observations were conducted under permission of the local authorities and in accordance with the ASAB guidelines for the treatment of animals in behavioural research (ASAB 2023).

## Conflicts of Interest

The authors declare no conflicts of interest.

## Supporting information


**Appendix S1:** ece372727‐sup‐0001‐Supinfo01.zip.

## Data Availability

Data are attached in the Supporting Information (Appendix S1).
