# Supplementary material for: A Direct Observation of Infanticide by a Female Free‐Ranging Dog (Canis familiaris) Supports the Resource Competition Hypothesis
Source: Ecol Evol. 2025 Dec 28;15(12):e72727. doi: 10.1002/ece3.72727 (PMC12998265; doi:10.1002/ece3.72727)

Supplement

**S1. Information on included females**

| **individual** | **DNA analysed?** | **group membership** | **litter date** | **dominance calculation date** | **elo score median** | **elo score q5 to q95** |
| --- | --- | --- | --- | --- | --- | --- |
| tofu | no* | black | 2023-04-22 | 2023-04-27 | 0.49 | -0.83 to 1.89 |
| tiny | yes | black | 2023-04-23 | 2023-04-27 | -1.35 | -2.08 to -0.61 |
| pain | yes | black | 2024-04-12 | 2024-03-27 | -0.98 | -1.20 to -0.70 |
| breeze | yes | black | 2024-05-30 | 2024-03-27 | -2.85 | -3.30 to -2.34 |
| mappy | yes | carpark | 2024-04-15 | 2024-03-27 | -2.85 | -1.01 to -0.22 |
| babouche | yes | carpark | 2024-04-15 | 2024-03-27 | -2.35 | -3.19 to -1.48 |
| ora | yes | cheese | 2023-03-01  2023-09-02  2024-02-29 | 2023-05-10  2023-09-02  2024-02-29 | 0.39  1.61  3.12 | -0.37 to 1.16  1.11 to 2.14  2.48 to 3.72 |
| maria | yes | cheese | 2023-03-17  2023-09-11  2024-04-09 | 2023-05-10  2023-09-02  2024-02-29 | 0.27  0.15  0.49 | -0.52 to 1.02  -0.09 to 0.62  0.42 to 0.56 |
| cruz | no* | cheese | 2023-09-07  2024-04-12 | 2023-09-02  2024-02-29 | -1.78  -3.61 | -2.37 to -1.29  -4.18 to -3.01 |
| shae | yes | fruit | 2022-12-14  2023-06-12  2024-04-14 | 2023-05-01  2023-06-12  2024-02-08 | 1.37  1.52  3.53 | 0.58 to 2.16  0.96 to 2.19  2.99 to 3.96 |
| bastama | no* | fruit | 2022-12-24  2023-07-01  2023-12-11 | 2023-05-01  2023-06-12  2023-09-27 | -1.21  -0.09  -0.98 | -2.11 to -0.25  -0.63 to 0.52  -1.21 to -0.71 |
| cottage | no | fruit | 2023-06-20 | 2023-06-12 | -1.48 | -2.40 to -0.56 |
| melaan | no* | fruit | 2023-07-23  2024-03-05 | 2023-06-12  2024-02-08 | -2.35  3.53 | -3.24 to -1.63  -3.50 to -2.47 |
| citrus | no* | fruit | 2023-09-27 | 2023-09-27 | 2.71 | 2.09 to 3.35 |
| papaya | yes | fruit | 2023-10-12 | 2023-09-27 | 1.12 | 0.60 to 1.71 |
| apollo | no* | fruit | 2024-02-08 | 2024-02-08 | -3.39 | -4.03 to -2.77 |
| fig | no | groom | 2023-08-12  2024-01-30 | 2023-08-12  2023-12-20 | 0.46  1.13 | 0.15 to 0.83  0.67 to 1.63 |
| nawir | yes | groom | 2023-08-26  2024-02-08 | 2023-08-12  2023-12-20 | -0.96  -0.10 | -1.36 to -0.52  -0.50 to 0.19 |
| anna | yes | groom | 2023-12-20 | 2023-12-20 | -1.03 | -0.41 to -0.61 |
| tache | no | before 25.06.2023: nova  after: market | 2023-05-08  2024-03-18 | 2023-05-11  2024-03-18 | 0.57  4.32 | -0.13 to 1.38  3.86 to 4.77 |
| lara | yes | before 25.06.2023: nova  after: market | 2023-04-09  2024-04-07 | 2023-05-11  2024-03-18 | 1.19  0.30 | 0.32 to 2.14  0.04 to 0.60 |
| jaws | yes | before 25.06.2023: nova  after: market | 2023-12-04 | 2023-12-04 | -2.88 | -3.67 to -2.39 |
| wasma | yes | before 25.06.2023: nova  after: market | 2023-12-30 | 2023-12-04 | -2.77 | -3.25 to -2.30 |
| nova | yes | nova | 2023-05-11  2023-11-13 | 2023-05-11  2023-11-13 | 1.64  2.99 | 0.75 to 2.67  2.41 to 3.58 |
| crof | yes | nova | 2023-05-11  2023-11-13 | 2023-05-11  2023-11-13 | 0.13  1.36 | -0.59 to 0.87  0.89 to 2.06 |
| pika | no | nova | 2023-06-21 | 2023-05-11 | -0.92 | -1.69 to -0.13 |

*kinship data from offspring available

**S2. Demographic kinship data for individuals without genetic data:**

Groom was observed grooming Fig, Nawir, Ora and two other sub-adults.

Pika and Tache were seen as pups, being groomed by Nova. Nursing was not directly observed since dogs were only seen briefly during scan observations, but Nova was the only lactating female in the group.

Cottage and Melaan are the same age and were seen near Shae as sub-adults. During the focal observations, as adults, they had frequent affiliative interactions with Shae. Kinship data shows that Melaan’s pup and Shae have a grandmother-grandchild relationship.

**S3. Elo-rating plots per group**


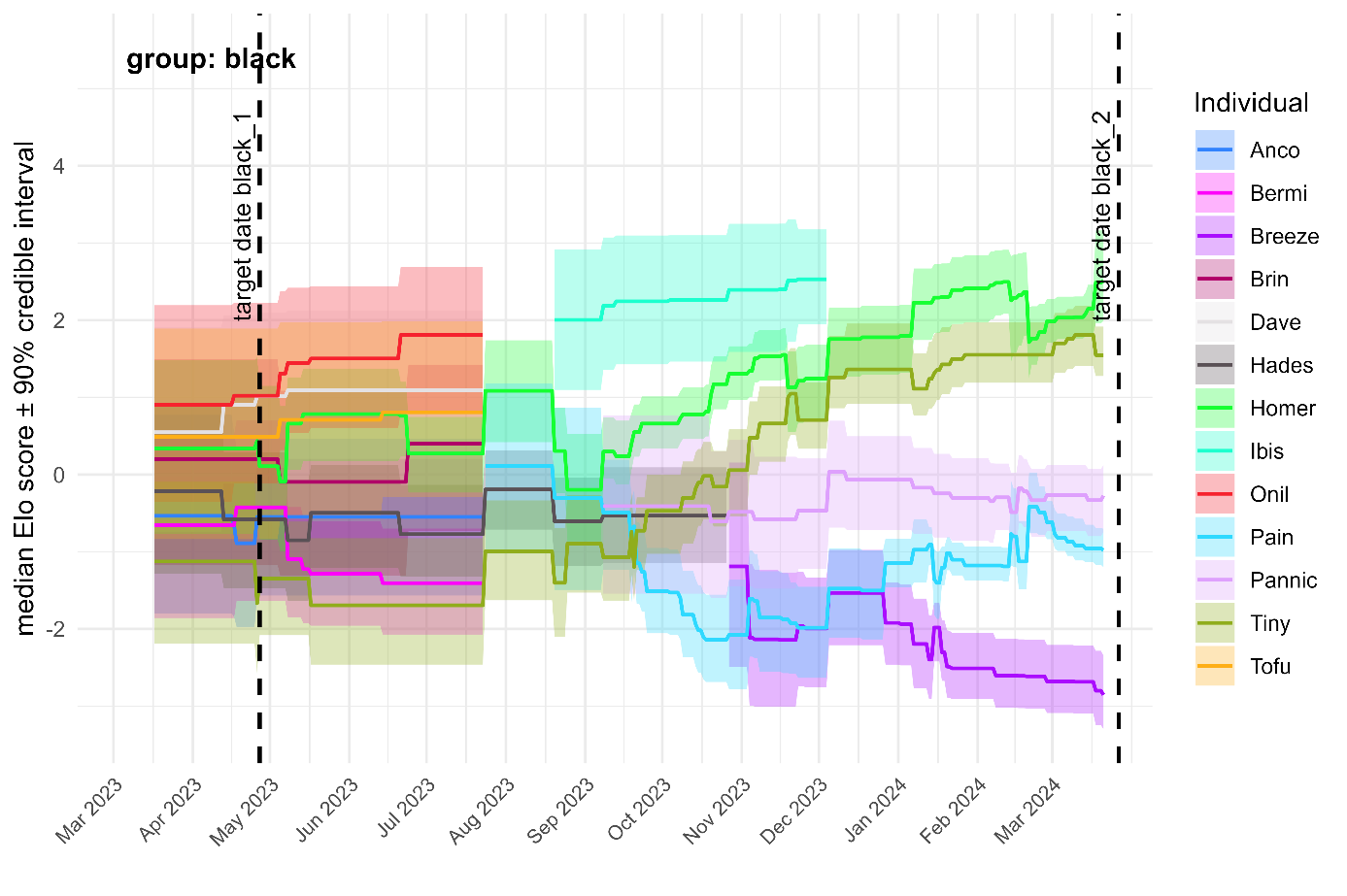


**Figure S3A.** Median Elo scores with 90% credible interval for the group ‘Black’, including all group members (including males, see ‘Methods’). The dashed line shows the target date for hierarchy calculation: April 27, 2023 and March 27, 2024. Females included in the birth sequence are: Tofu and Tiny in black_1, Breeze and Pain in black_2.


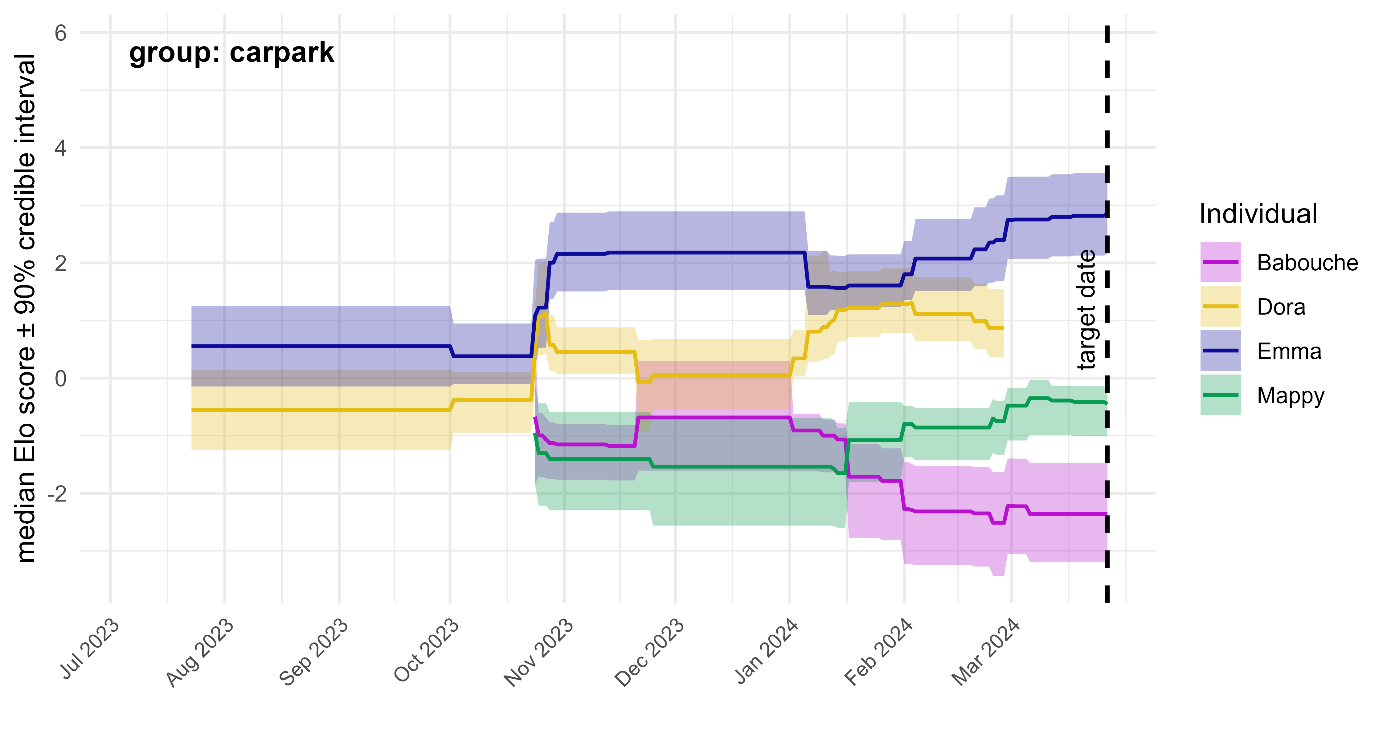


**Figure S3B.** Median Elo scores with 90% credible interval for the group ‘Carpark’, including all female group members. The dashed lines show the target dates for hierarchy calculation: March 27, 2024. Females included in the birth sequence are: Mappy and Babouche.


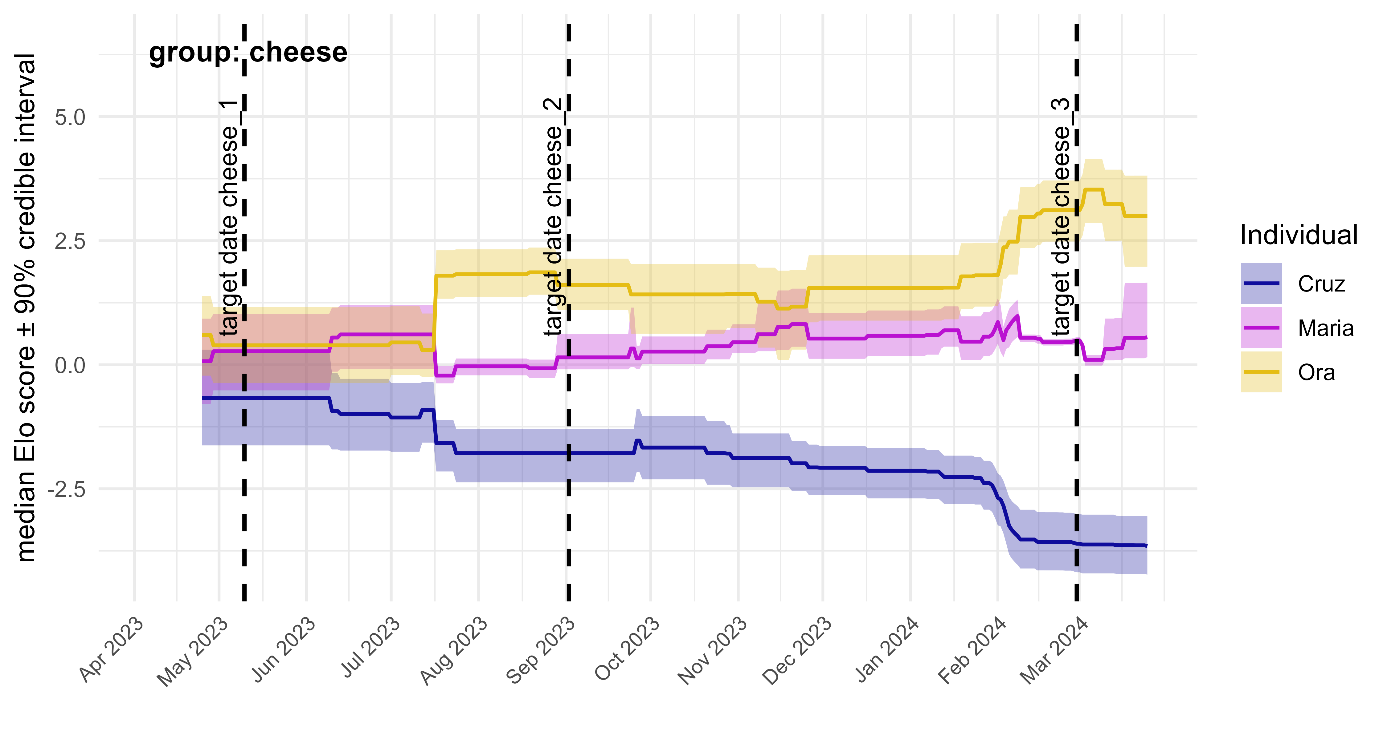


**Figure S3C.** Median Elo scores with 90% credible interval for the group ‘Cheese’, including all female group members. The dashed lines show the target dates for hierarchy calculation: May 10, 2023; September 2, 2023 and February 29, 2024. Females included in the birth sequence are: Ora and Maria in cheese_1; Ora, Maria and Cruz in cheese_2 and cheese_3.


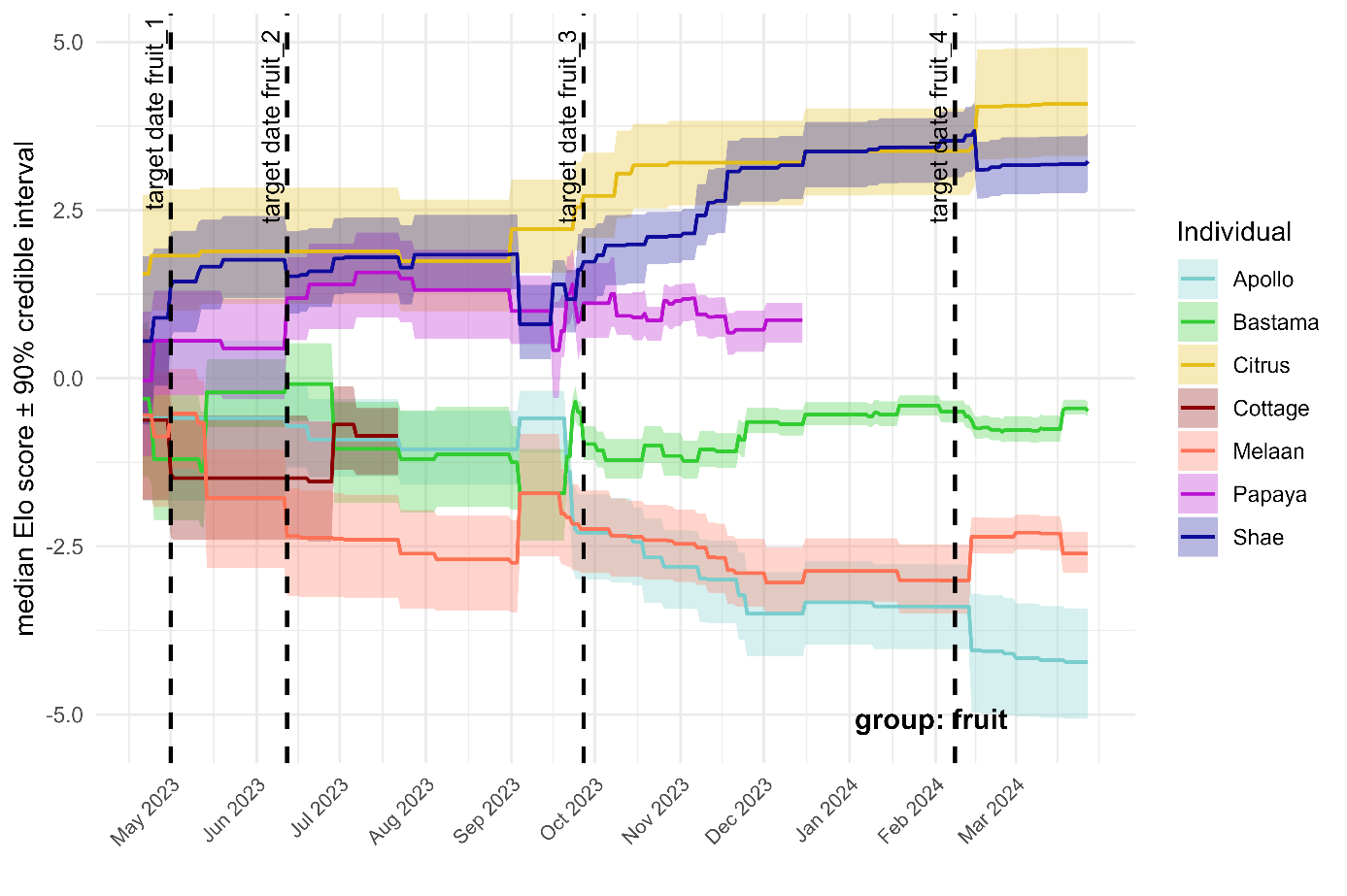


**Figure S3D.** Median Elo scores with 90% credible interval for the group ‘Fruit’, including all female group members. The dashed lines show the target dates for hierarchy calculation: May 1, 2023; June 12, 2023; September 27, 2023 and February 8, 2024. Females included in the birth sequence are: Bastama and Shae in fruit_1; Bastama, Cottage, Melaan and Shae in fruit_2; Bastama, Citrus and Papaya in fruit_3 and Apollo, Melaan and Shae in fruit_4.


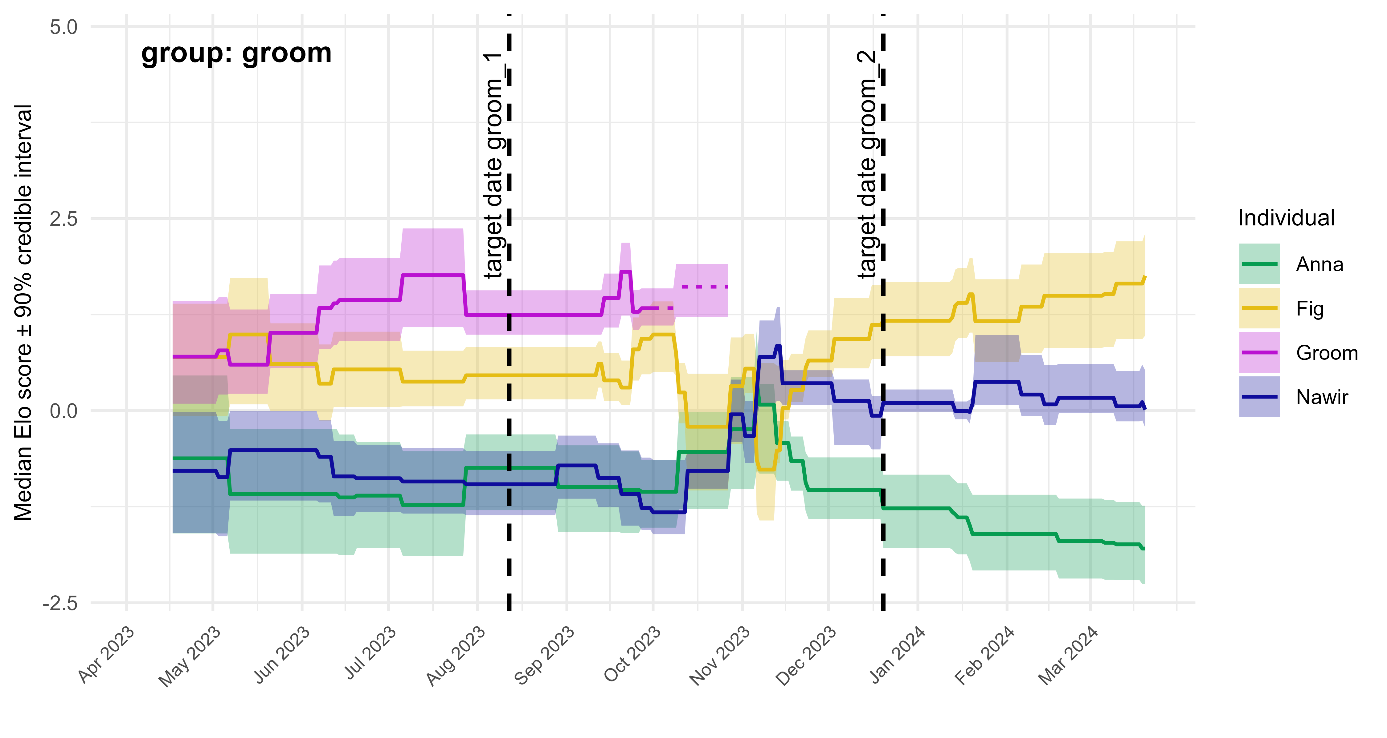


**Figure S3E.** Median Elo scores with 90% credible interval for the group ‘Groom’, including all female group members. The dashed lines show the target dates for hierarchy calculation: August 12, 2023 and December 20, 2023. Females included in the birth sequence are: Fig and Nawir in groom_1, Fig, Nawir and Anna in groom_2.


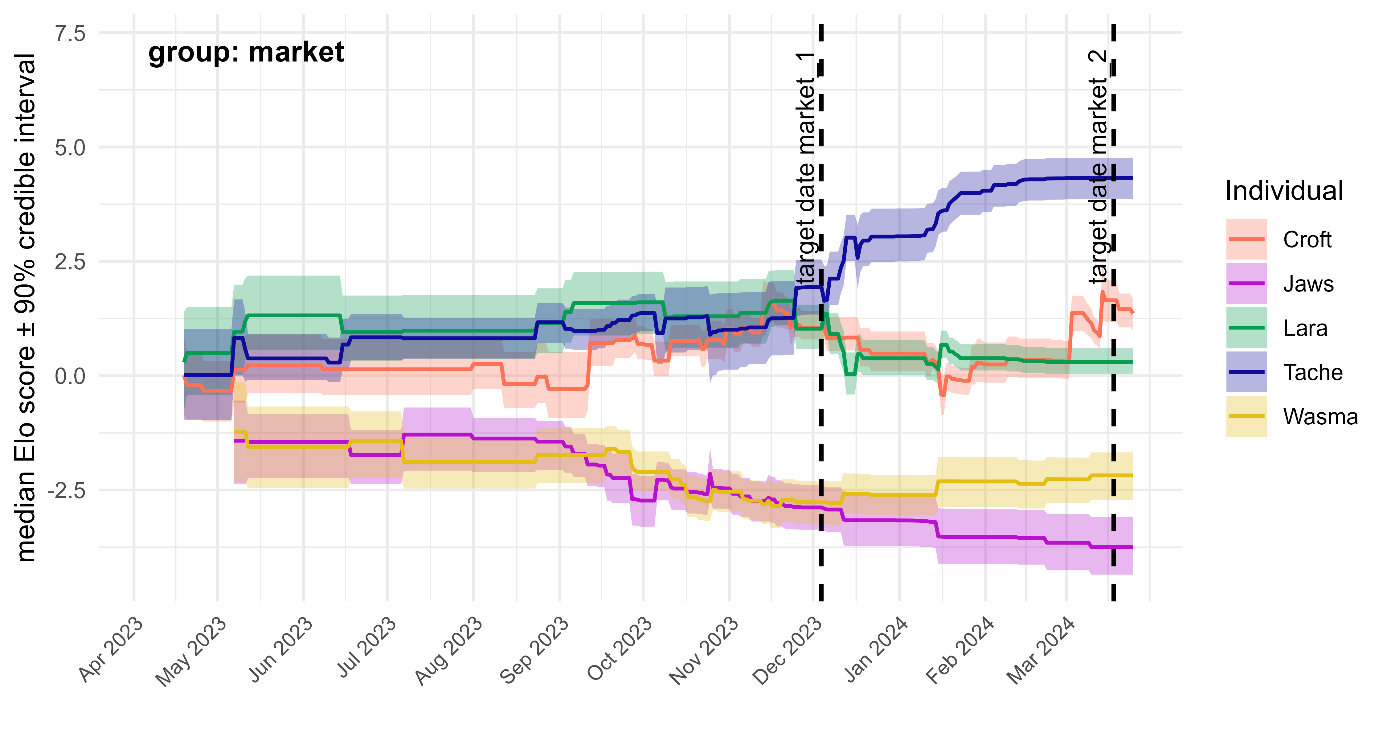


**Figure S3F.** Median Elo scores with 90% credible interval for the group ‘Market’, including all female group members. The dashed lines show the target dates for hierarchy calculation: December 12, 2023 and March 18, 2024. Females included in the birth sequence are: Jaws and Wasma in market_1, Lara and Tache in market_2. The ‘Market’ pack split off from the ‘Nova’ pack in July 2023.


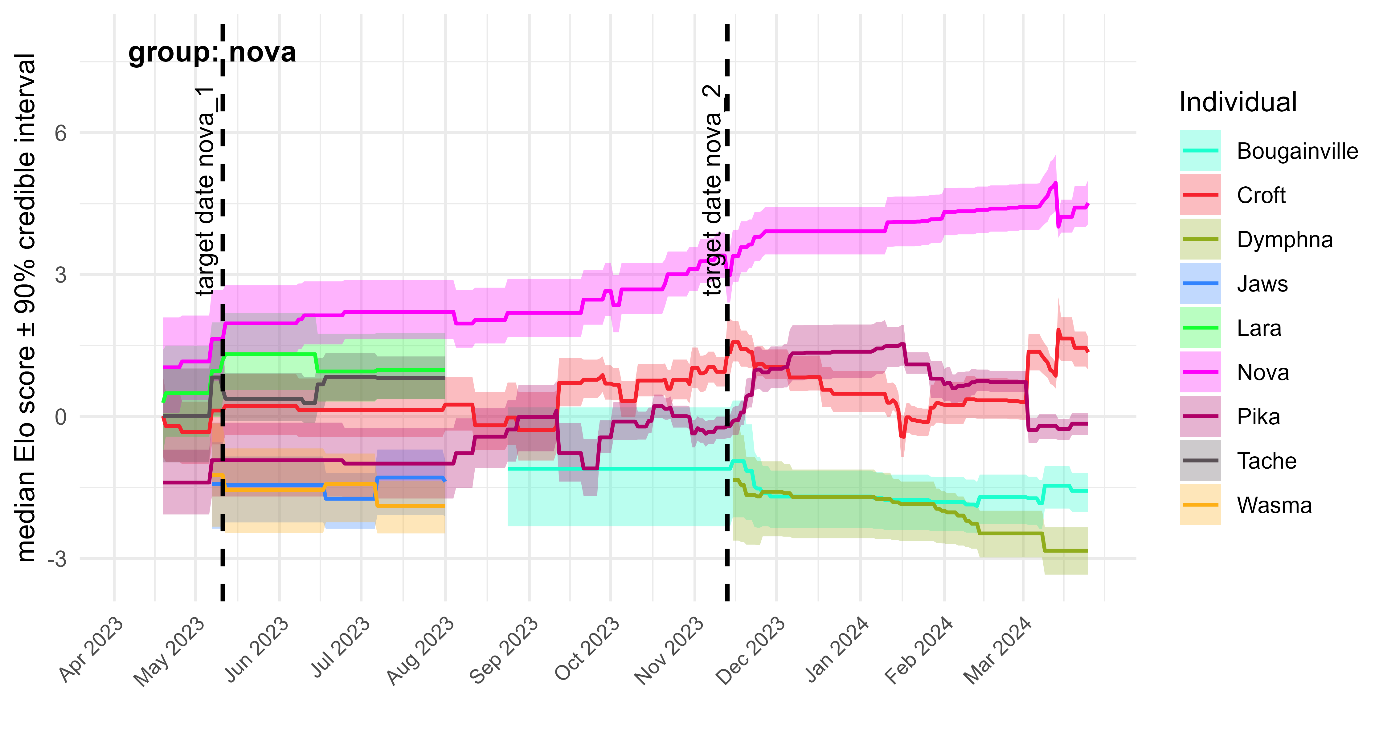


**Figure S3G.** Median Elo scores with 90% credible interval for the group ‘Nova’, including all female group members. The dashed lines show the target dates for hierarchy calculation: May 11, 2023 and November 13, 2023. Females included in the birth sequence are: Croft, Lara, Nova and Pika in nova_1, Croft and Nova in nova_2.

**S4. Images of free-ranging dogs chasing wild boars, near the location where the infanticide took place.**


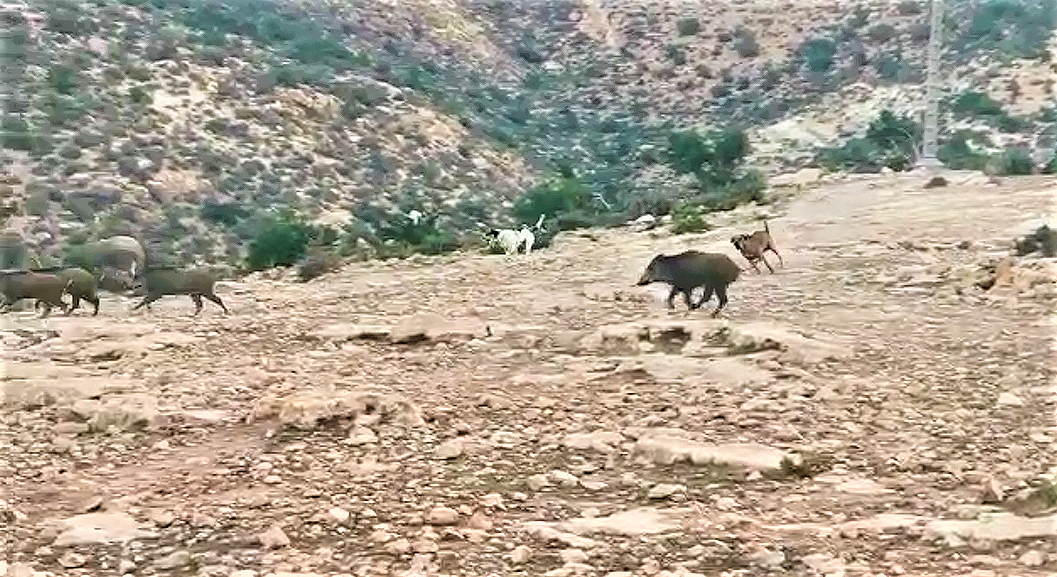

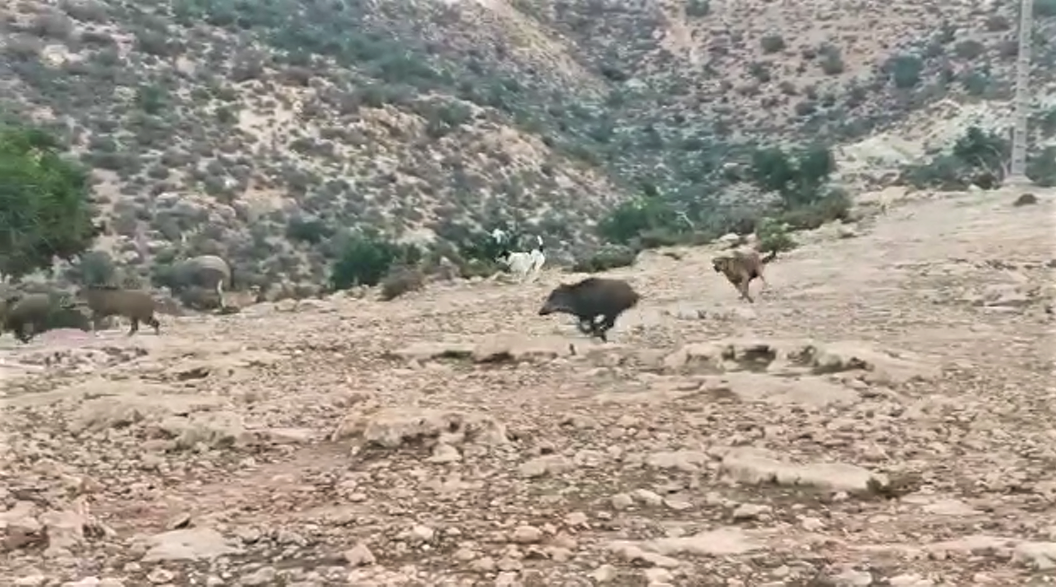

Supplement: Supplementary file 2 — Appendix S1: ece372727‐sup‐0001‐Supinfo01.zip. [file ECE3-15-e72727-s001.zip › Vanderheyden_etal_supplement_20250817.docx]
